# Supplementary material for: Interaction of the mitochondrial calcium/proton exchanger TMBIM5 with MICU1
Source: Commun Biol. 2025 Sep 19;8:1348. doi: 10.1038/s42003-025-08839-6 (PMC12449474; doi:10.1038/s42003-025-08839-6)
Supplement: Supplementary file 2 — Supplementary Information [file 42003_2025_8839_MOESM2_ESM.pdf]

Supplemental Figures

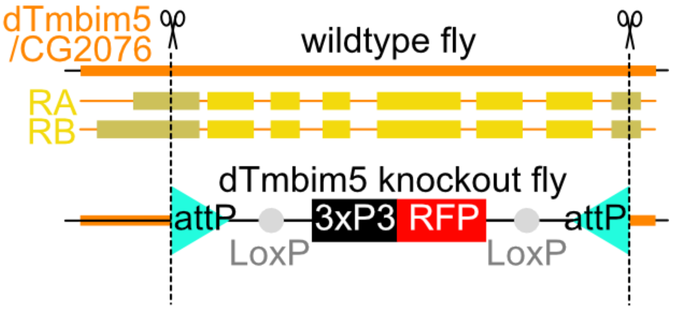

Figure S1. Generation of *dTmbim5* knockout flies

Knockout *dTmbim5* (*CG2076*) flies were generated using CRISPR/Cas9-mediated homology-directed repair deleting a 1,460-bp fragment (-36 nt to +1,424 nt from the ATG of *CG2076*) and replacing with a 3xP3-RFP cassette flanked by attP sites. RA and RB indicate two different transcript variants.

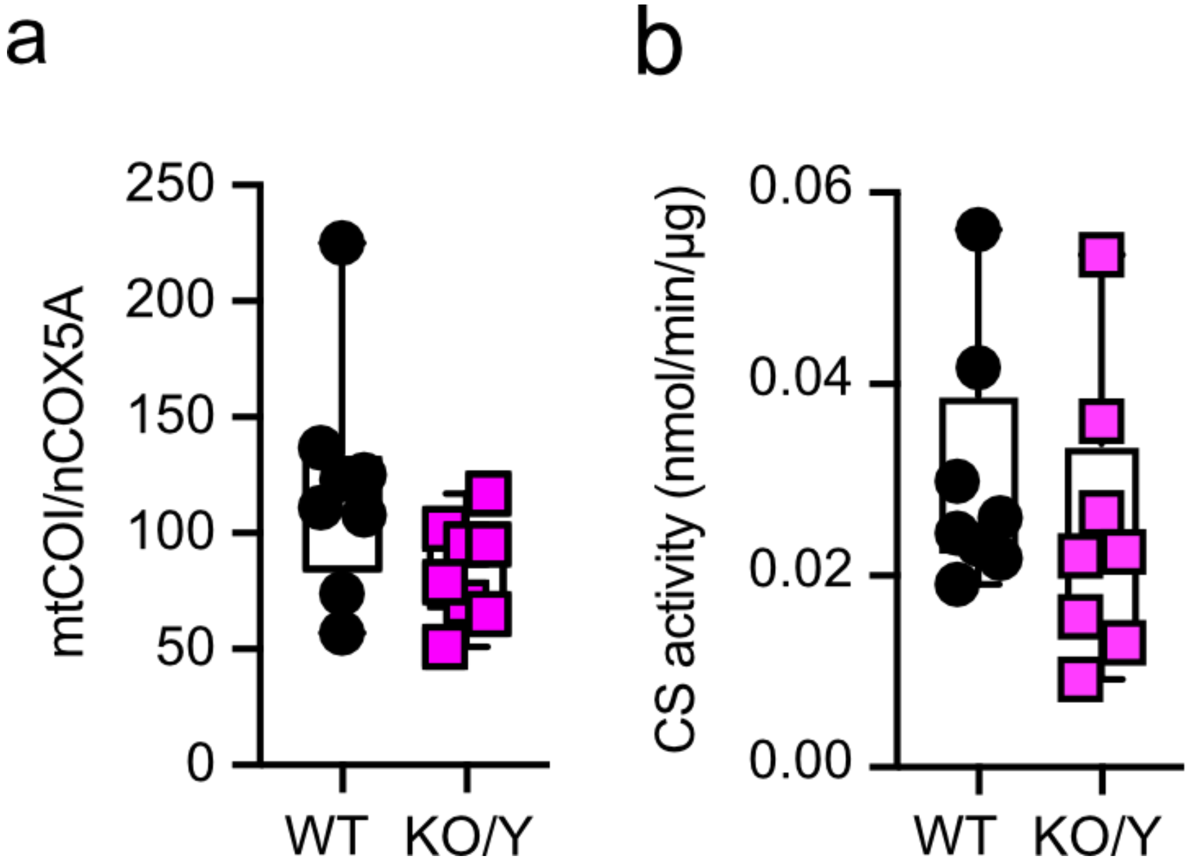

Figure S2. Assessment of mitochondrial content in *Tmbim5* knockout flies reveals no significant alterations in mitochondrial mass (a) Mitochondrial DNA copy number was quantified by comparing the relative abundance of mitochondrially encoded *mtCOI* (Drosophila ortholog of human cytochrome c oxidase subunit I) to nuclear-encoded *nCOX5A* (Drosophila ortholog of human cytochrome c oxidase subunit 5A) using quantitative PCR analysis of total DNA extracts. **(b)** Citrate synthase (CS) activity was measured in mitochondrial preparations. Citrate synthase is a nuclear-encoded enzyme localized in the mitochondrial matrix whose activity remains stable under various metabolic conditions, making it an ideal reference enzyme for normalizing mitochondrial content across experimental conditions. Data are represented as box and whisker plots showing the interquartile range (25th to 75th percentile) with the median indicated by a horizontal line. Whiskers extend from minimum to maximum values. WT represents wild-type (w^{1118}) flies, while KO/Y indicates hemizygous *Tmbim5* knockout males. Statistical analysis was performed using unpaired t-tests which revealed no significant changes.

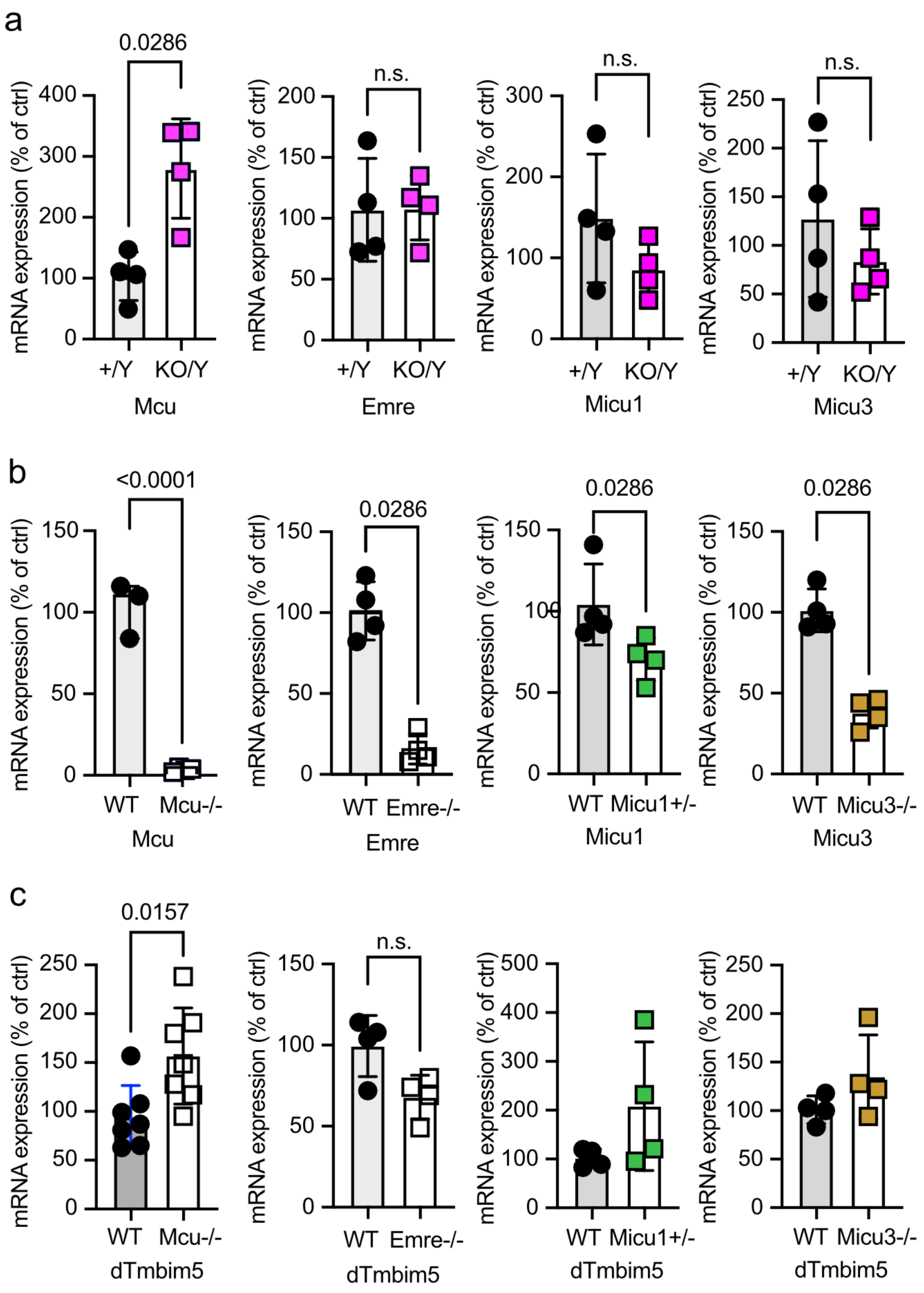
a
0.0286
n.s.
n.s.
n.s.
mRNA expression (% of ctrl)
+/Y
KO/Y
Mcu
Emre
Micu1
Micu3
b
<0.0001
0.0286
0.0286
0.0286
WT
Mcu-/-
Emre-/-
Micu1+/-
Micu3-/-
c
0.0157
n.s.
dTmbim5

Figure S3. Reciprocal transcriptional regulation between *Tmbim5* and *Mcu* in *Drosophila melanogaster*. (a) Quantitative PCR analysis of mitochondrial calcium uniporter complex components in *Tmbim5* knockout (KO/Y) male flies compared to wildtype (+/Y) controls. Data demonstrate significant upregulation of *Mcu* mRNA levels while *Emre*, *Micu1*, and *Micu3* transcript abundance remains unchanged. Expression values were normalized to *Rp49* as an endogenous reference gene. **(b)** Validation of genetic models through transcript quantification in respective knockout lines: *Mcu* knockout (*Mcu-/-*), *Emre* knockout (*Emre-/-*), *Micu1* heterozygous knockout (*Micu1+/-*), and *Micu3* knockout (*Micu3-/-*). Quantitative PCR confirms significant reduction of target transcripts in each genetic background compared to wildtype controls. **(c)** Quantitative PCR analysis of *Tmbim5* expression across mitochondrial calcium uniporter complex mutant backgrounds. Data reveal significant upregulation of *Tmbim5* transcript levels specifically in *Mcu-/-* flies, with no significant changes observed in *Emre-/-*, *Micu1+/-*, or *Micu3-/-* genetic backgrounds. Data across all panels are presented as bar graphs showing mean ± standard deviation. Each data point represents the mean value derived from pooled RNA of five 4-7-day-old flies. Statistical significance was determined using the Mann-Whitney test with *p* values indicated.
