# Supplementary material for: Interaction of the mitochondrial calcium/proton exchanger TMBIM5 with MICU1
Source: Commun Biol. 2025 Sep 19;8:1348. doi: 10.1038/s42003-025-08839-6 (PMC12449474; doi:10.1038/s42003-025-08839-6)
Supplement: Supplementary file 3 — Description of Additional Supplementary Files [file 42003_2025_8839_MOESM3_ESM.pdf]

Description of Additional Supplementary Files

File name: Supplementary data

Description: All data and uncropped immunoblots
