# Supplementary material for: Interaction of the mitochondrial calcium/proton exchanger TMBIM5 with MICU1
Source: Commun Biol. 2025 Sep 19;8:1348. doi: 10.1038/s42003-025-08839-6 (PMC12449474; doi:10.1038/s42003-025-08839-6)
Supplement: Supplementary file 5 — Reporting summary [file 42003_2025_8839_MOESM5_ESM.pdf]

nature portfolio

Corresponding author(s): *Double-anonymous peer review submissions: write DAPR and your manuscript number here instead of author names.*

Last updated by author(s): Jul 8, 2025

Reporting Summary

Nature Portfolio wishes to improve the reproducibility of the work that we publish. This form provides structure for consistency and transparency in reporting. For further information on Nature Portfolio policies, see our Editorial Policies and the Editorial Policy Checklist.

Statistics

For all statistical analyses, confirm that the following items are present in the figure legend, table legend, main text, or Methods section.

n/a	Confirmed	
☐	☒	The exact sample size (n) for each experimental group/condition, given as a discrete number and unit of measurement
☐	☒	A statement on whether measurements were taken from distinct samples or whether the same sample was measured repeatedly
☐	☒	The statistical test(s) used AND whether they are one- or two-sided<br>*Only common tests should be described solely by name; describe more complex techniques in the Methods section.*
☒	☐	A description of all covariates tested
☐	☒	A description of any assumptions or corrections, such as tests of normality and adjustment for multiple comparisons
☐	☒	A full description of the statistical parameters including central tendency (e.g. means) or other basic estimates (e.g. regression coefficient) AND variation (e.g. standard deviation) or associated estimates of uncertainty (e.g. confidence intervals)
☒	☐	For null hypothesis testing, the test statistic (e.g. F, t, r) with confidence intervals, effect sizes, degrees of freedom and P value noted<br>*Give P values as exact values whenever suitable.*
☒	☐	For Bayesian analysis, information on the choice of priors and Markov chain Monte Carlo settings
☒	☐	For hierarchical and complex designs, identification of the appropriate level for tests and full reporting of outcomes
☒	☐	Estimates of effect sizes (e.g. Cohen's d, Pearson's r), indicating how they were calculated

Our web collection on statistics for biologists contains articles on many of the points above.

Software and code

Policy information about availability of computer code

Data collection	Data were collected using standard instrument acquisition software, including Nikon NIS-Elements AR 4.51.00 64-bit (Nikon) for SIM image acquisition, the Spark Multimode Microplate Reader (Tecan) for absorbance and ATP measurements, and Tecnai 12 (Thermo Fisher Scientific) for transmission electron microscopy. No custom code or open-source data collection tools were used.
Data analysis	Data analysis was performed using GraphPad Prism version 10.2.3 (GraphPad Software), ImageJ (NIH), and Nikon NIS-Elements AR 4.51.00 64-bit (Nikon). No custom algorithms or code were used.

For manuscripts utilizing custom algorithms or software that are central to the research but not yet described in published literature, software must be made available to editors and reviewers. We strongly encourage code deposition in a community repository (e.g. GitHub). See the Nature Portfolio guidelines for submitting code & software for further information.

Data

Policy information about availability of data

All manuscripts must include a data availability statement. This statement should provide the following information, where applicable:

- Accession codes, unique identifiers, or web links for publicly available datasets
- A description of any restrictions on data availability
- For clinical datasets or third party data, please ensure that the statement adheres to our policy

All data supporting the findings of this study are available within the article and its supplementary information files. Additional raw data are available from the corresponding author upon reasonable request.

Research involving human participants, their data, or biological material

Policy information about studies with human participants or human data. See also policy information about sex, gender (identity/presentation), and sexual orientation and race, ethnicity and racism.

Reporting on sex and gender	This study did not involve human participants, human data, or human tissue. Not applicable.
Reporting on race, ethnicity, or other socially relevant groupings	Not applicable
Population characteristics	Not applicable
Recruitment	Not applicable
Ethics oversight	Not applicable

Note that full information on the approval of the study protocol must also be provided in the manuscript.

Field-specific reporting

Please select the one below that is the best fit for your research. If you are not sure, read the appropriate sections before making your selection.

☒ Life sciences ☐ Behavioural & social sciences ☐ Ecological, evolutionary & environmental sciences

For a reference copy of the document with all sections, see nature.com/documents/nr-reporting-summary-flat.pdf

Life sciences study design

All studies must disclose on these points even when the disclosure is negative.

Sample size	No formal sample size calculation was performed. Sample sizes were based on commonly accepted standards in the field and our previous experience with similar experiments, ensuring adequate power to detect biologically relevant differences. Sample sizes and replicates are provided in the figure legends and Methods section.
Data exclusions	No data were excluded from the analyses unless otherwise stated in the Methods. All data points are shown in the figures. No pre-established exclusion criteria were used.
Replication	All key experiments were independently replicated at least three times, with consistent results. Replication details, including the number of independent experiments and biological replicates, are provided in the figure legends and Methods section.
Randomization	Randomization was not applicable, as group allocation was determined by genotype or experimental treatment.
Blinding	Investigators were not blinded to group allocation during data collection or analysis, as group identity (e.g., genotype or treatment) was apparent from the experimental setup and necessary for proper handling of samples. However, objective, quantitative methods (e.g., automated imaging analysis, plate reader measurements) were used where possible to minimize potential bias.

Reporting for specific materials, systems and methods

We require information from authors about some types of materials, experimental systems and methods used in many studies. Here, indicate whether each material, system or method listed is relevant to your study. If you are not sure if a list item applies to your research, read the appropriate section before selecting a response.

Materials & experimental systems

n/a	Involved in the study
☐	☒ Antibodies
☐	☒ Eukaryotic cell lines
☒	☐ Palaeontology and archaeology
☐	☒ Animals and other organisms
☒	☐ Clinical data
☒	☐ Dual use research of concern
☒	☐ Plants

Methods

n/a	Involved in the study
☒	☐ ChIP-seq
☒	☐ Flow cytometry
☒	☐ MRI-based neuroimaging

Antibodies

Antibodies used

The following antibodies were used in this study (supplier, catalog number, RRID, dilution):
- Rabbit polyclonal anti-TMBIM5 (Proteintech, Cat#16296-1-AP; RRID:AB_2111275; 1:1,000)
- Rabbit polyclonal anti-MCU (Millipore Sigma, Cat#HPA016480; RRID:AB_2071893; 1:500)
- Rabbit polyclonal anti-MICU1 (Merck, Cat#HPA037479; RRID:AB_2675495; 1:500)
- Rabbit polyclonal anti-MICU2 (Abcam, Cat#ab101465; RRID:AB_10711219; 1:500)
- Rabbit polyclonal anti-alpha-LETM1 (Thermo Fisher, Cat#PA5-22233; RRID:AB_11152727; 1:500)
- Rabbit polyclonal anti-EMRE (Santa Cruz, Cat#sc-86337; RRID:AB_2250685; 1:200)
- Rabbit anti-HA (Abcam, Cat#ab9110-100; RRID:AB_307019; 1:500)
- Mouse monoclonal anti-alpha-actin (Merck chemicals, Cat#MAB1501; RRID:AB_2223041; 1:1,000)
- Anti-mouse IgG (H+L) (DyLight 680 Conjugate, Cell Signaling, Cat#5470S; RRID:AB_10696895, 1:10,000)
- Anti-rabbit IgG (H+L) (DyLight 800 Conjugate, Cell Signaling, Cat#5151S; RRID:AB_10697505, 1:10,000)

Validation

All primary antibodies used are commercially available and widely validated for immunoblotting in the relevant species. Validation statements are provided by the manufacturers on their respective websites. In addition, expected band sizes were observed, and specificity was confirmed by the use of knockout.

Eukaryotic cell lines

Policy information about cell lines and Sex and Gender in Research

Cell line source(s)

HEK293 wild-type and TMBIM5 knockout (KO) cell lines were obtained from Engineered Cells. Additional HEK293-based cell lines were kindly provided by Dr. Kevin Foskett (University of Pennsylvania). HEK293 cells are derived from a female human embryo.

Authentication

The identity of HEK293 cell lines was confirmed by their characteristic morphology and growth properties, as well as by genotyping for TMBIM5 knockout. No formal STR profiling was performed.

Mycoplasma contamination

All HEK293 cell lines were routinely tested for mycoplasma contamination using the MycoStrip™ Mycoplasma Detection Kit (InvivoGen, Cat# rep-mys-20, Lot# 51862225) and found to be negative prior to experiments.

Commonly misidentified lines
(See ICLAC register)

HEK293 is not listed as a commonly misidentified cell line in the ICLAC register.

Animals and other research organisms

Policy information about studies involving animals; ARRIVE guidelines recommended for reporting animal research, and Sex and Gender in Research

Laboratory animals

Drosophila melanogaster were used for all animal experiments. Details of strains, genotypes, and ages of flies are provided in the Methods section. Flies were maintained at 25°C on standard cornmeal-agar medium.

Wild animals

This study did not involve wild animals.

Reporting on sex

Male Drosophila were used for most experiments, as the Tmbim5 gene is located on the X chromosome and for genetic crossing purposes. The sex of flies used in each experiment is specified in the Methods section.

Field-collected samples

This study did not involve samples collected from the field.

Ethics oversight

Experiments involving Drosophila melanogaster were performed in accordance with institutional and national guidelines for the care and use of animals in research. Invertebrate research using Drosophila does not require formal ethical approval at our institution.

Note that full information on the approval of the study protocol must also be provided in the manuscript.

Plants

Seed stocks	Not applicable. This study did not involve any novel plant genotypes.
Novel plant genotypes	Not applicable. This study did not use any plant seed stocks.
Authentication	Not applicable. No plant materials were used in this study.
